# University-Based Animal Welfare Organizations in Ecuador: A National One Health Perspective

**DOI:** 10.3390/ani16182885

**Published:** 2026-09-14

**Authors:** Ángel Sebastián Rodríguez-Pazmiño, Sarita Doménica Bautista-Arcentales, Miguel Angel Garcia-Bereguiain, Solon Alberto Orlando

**Affiliations:** 1 One Health Research Group, Universidad de Las Américas, Quito 170124, Ecuador; sarita.bautista@udla.edu.ec (S.D.B.-A.); miguel.garcia.bereguiain@udla.edu.ec (M.A.G.-B.); 2 Facultad de Ingeniería, Arquitectura y Ciencias de la Naturaleza, Campus La Costa, Universidad Ecotec, Km 13.5 Samborondón, Samborondón EC092302, Ecuador; sorlando@ecotec.edu.ec

**Keywords:** dog and cat population management, animal welfare, One Health, universities, Ecuador

## Abstract

University-based animal welfare organizations are grassroots actors involved in managing free-roaming dog and cat populations, yet their role remains poorly characterized, particularly in low- and middle-income countries. This study aimed to identify and describe animal welfare organizations operating within universities in Ecuador and to analyze their potential contributions within a One Health framework. A cross-sectional survey of 63 universities was conducted using publicly available information from institutional websites and social media platforms, complemented by semi-structured interviews with representatives of identified organizations. Organizations with evidence of activity through 2026 and a focus on dogs or cats were included. A total of 14 animal welfare organizations from 12 universities were identified, representing 19.0% of the universities analyzed. Most organizations (71.4%) were located in public universities, and 35.7% were associated with veterinary-related programs. Framework-informed analysis of interview data (12 out of 14 organizations interviewed) revealed four key dimensions of organizational functioning: capacity and governance, animal care and population management, public health and community engagement, and institutional integration. Reported activities included animal rescue, sterilization campaigns, adoption initiatives, and community outreach, aligning with internationally recommended strategies for humane population management. Several organizations have extended their reach beyond campus boundaries through free veterinary intervention campaigns in rural communities, partnerships with municipal authorities, and collaboration with civil society networks, reflecting a broader public health role consistent with One Health principles. Despite their potential, these organizations face structural challenges, including high volunteer turnover, the absence of direct institutional funding, and limited formal integration into governmental frameworks. Institutional support, where present, is predominantly material rather than financial, placing the burden of sustainability on self-management mechanisms. Nonetheless, their embeddedness within academic environments positions them as strategic platforms for interdisciplinary collaboration, experiential training, and community engagement. This study highlights the need to recognize and integrate university-based animal welfare organizations into broader animal population management and public health strategies. Strengthening partnerships between academia, government, and civil society could enhance both animal welfare outcomes and zoonotic disease surveillance efforts in Ecuador and other low- and middle-income countries.

## Simple Summary

Free-roaming dogs and cats are a common sight in cities across Ecuador and many other countries, and their management poses challenges for animal welfare, public health, and urban coexistence. Universities can play an important role in addressing these challenges, as they bring together students, academic expertise, and community engagement capacity. However, little is known about the animal welfare organizations that operate within universities, particularly in lower-income countries. This study identified and described all university-based animal welfare organizations active in Ecuador, using publicly available information from university websites and social media, complemented by interviews with representatives of the identified groups. A total of 14 organizations were found across 12 universities, representing nearly one in five of the country’s recognized higher education institutions. These groups carry out a wide range of activities, including rescuing animals, sterilization campaigns, adoptions, veterinary care, and community education. Despite operating with very limited resources and no direct financial support from their universities, they make meaningful contributions to animal care and community engagement. The study highlights the potential of these organizations as partners in broader efforts to improve animal welfare and public health, and calls for greater institutional recognition and support.

## 1. Introduction

Free-roaming dogs and cats represent a persistent challenge at the intersection of animal welfare, public health, and environmental management worldwide. In low- and middle-income countries (LMICs), unmanaged dog and cat populations are associated with the transmission of zoonotic diseases, including rabies and other parasitic and bacterial infections, as well as environmental contamination and community conflict [1,2,3]. Addressing these challenges requires comprehensive and sustained strategies for dog and cat population management that integrate animal welfare, public health, and community-based approaches [4,5,6].

Population management encompasses a range of coordinated interventions, including sterilization, vaccination, access to veterinary care, promotion of responsible ownership, sheltering, and community education. Within this broader framework, sterilization is an important component, but its effectiveness depends on integration with complementary actions and sustained implementation over time [4,5,7]. From a One Health perspective, these interventions contribute to interconnected human, animal, and environmental health outcomes by reducing disease transmission risks, improving animal health, and promoting safer human–animal interactions [8]. Additionally, the management of dog and cat populations should be viewed not only as an animal-welfare intervention but also as a public-health and environmental strategy.

Despite the recognized role of civil society, one category of key actors remains largely undocumented in the scientific literature: university-based animal welfare organizations, particularly those led or coordinated by students or from veterinary schools. Universities represent strategic environments for One Health implementation, as they integrate academic expertise, training programs in health-related disciplines, research infrastructure, and community engagement platforms [8]. Initiatives embedded within these institutions may therefore contribute to multiple dimensions of population management, including service delivery, community engagement, and data generation for research and surveillance.

However, the prevalence, structure, and activities of university-affiliated animal welfare organizations remain poorly characterized, particularly in LMIC contexts. Ecuador provides a relevant case study, given its diverse higher education system comprising 63 universities distributed nationwide [9,10]. Free-roaming dogs and cats are common in urban and peri-urban areas, and population management efforts are typically implemented through collaborations among municipalities, veterinarians, and non-governmental organizations. Nevertheless, the extent to which universities—and especially student-led organizations—participate in or influence these efforts has not been systematically assessed.

Understanding the role of university-based organizations is important for several reasons. First, universities may serve as relatively stable institutional platforms that can support sustained animal welfare and population management initiatives. Second, they may facilitate the integration of research and surveillance components into these efforts, strengthening the evidence base for decision-making. Third, student participation may foster early engagement with One Health principles, contributing to workforce development in animal and public health sectors. These advantages may be further strengthened when organizations are embedded within veterinary education programs.

Internationally, universities and campus-based animal welfare initiatives have been recognized as important settings for managing free-roaming companion animals and promoting community engagement, education, and One Health approaches. In the United States, university-affiliated initiatives such as the University of Florida’s community cat programs and the Stanford Cat Network have demonstrated the role that campus-based groups can play in sterilization, adoption, and population monitoring efforts [11,12]. Similar experiences have been reported in other countries, including Australia, where campus cat management programs have been evaluated at the University of New South Wales [13], as well as in Brazil, Lebanon, and South Africa, where university- or community-linked initiatives have contributed to humane population management and public awareness [14,15,16]. Most published campus-based studies have focused primarily on cat management, reflecting the prominence of free-roaming cat populations in many university environments. In contrast, the present study identified that university-based animal welfare organizations in Ecuador commonly address both dogs and cats, with a notable emphasis on dog-related activities. This pattern may reflect the high visibility of free-roaming dogs in Ecuadorian urban and peri-urban settings, as well as the priorities adopted by local animal welfare organizations in response to community needs. By documenting these organizations at a national level, this study contributes evidence from a low- and middle-income country context and expands the international discussion on the role of universities in humane animal population management and One Health initiatives.

The present study aims to (1) systematically identify universities in Ecuador hosting animal welfare organizations using publicly available information, (2) characterize their structure, activities, and level of institutional support, and (3) explore their current and potential contributions to dog and cat population management within a One Health framework, including insights derived from structured interviews. By providing a national-level overview of these organizations, this study seeks to inform discussions on institutional capacity, community engagement, and the integration of academic actors into population management strategies in LMIC settings.

## 2. Methods

### 2.1. Study Design

This study was designed as a national, cross-sectional, exploratory study that combined systematic institutional mapping and semi-structured interviews to characterize university-based animal welfare organizations in Ecuador. The study aimed to assess their structure, activities, and institutional integration, and to explore their current and potential contributions to the management of free-roaming dogs and cats within a One Health framework. Specifically, the study addressed three objectives: (1) to systematically identify universities in Ecuador hosting animal welfare or animal protection organizations; (2) to characterize the structure, activities, and institutional support of these organizations; and (3) to analyze their current activities and potential contributions to the management of free-roaming dog and cat populations, including but not limited to sterilization-based approaches.

### 2.2. Identification of Universities and Institutional Mapping

According to the Consejo de Aseguramiento de la Calidad de la Educación Superior (CACES), Ecuador has 63 officially recognized universities and polytechnic schools [9]. A comprehensive list of these institutions was compiled using publicly available information from official regulatory and institutional sources (see Supplementary Material S1). Each university was systematically screened to identify whether it had animal welfare or animal protection organizations. This process included reviewing official university websites, student association directories, institutional news sections, and publicly accessible social media platforms linked to each institution. When relevant initiatives were identified, available contact information was collected from institutional pages or official social media accounts. This systematic screening enabled the identification of universities hosting animal welfare organizations and the estimation of their national distribution and prevalence. Searches were conducted in Spanish, reflecting Ecuador’s institutional and social media landscape. The keywords used included *bienestar animal*, *protección animal*, *rescate animal*, *animales universitarios*, *perros campus*, *gatos campus*, and *club animal*, applied in combination with the name of each university. An organization was considered eligible for inclusion if it met all of the following criteria: (1) it was identifiably associated with a university or polytechnic school recognized by CACES; (2) it demonstrated a primary focus on dogs, cats, or both; and (3) it showed evidence of activity through 2026, based on posts, publications, or institutional records accessible at the time of the search. Organizations for which no evidence of recent activity could be identified were excluded, regardless of whether historical records of their existence were found.

To enhance the reliability of the search process, a random sample of universities was independently screened by a second researcher (S.D.B.A.) to verify the completeness and consistency of the identification process. Any discrepancies in the identification of eligible organizations were resolved by consensus.

### 2.3. Participant Recruitment and Data Collection

Representatives of identified university-based animal welfare organizations were contacted via email or social media. Organizations that confirmed they were actively operating were invited to participate in a semi-structured interview. Interviews were conducted online via Google Meet (Google LLC, Mountain View, CA, USA) using a semi-structured interview guide (Supplementary Material S2) to ensure consistency across participants. The guide included questions on: year of foundation, organizational structure and leadership, number of active members, types of activities conducted (e.g., sterilization, adoption, animal care, educational outreach), number of animals assisted, sterilizations performed, and adoptions facilitated (when available), relationship with the host university (e.g., institutional recognition, logistical support, academic involvement), sources of funding, perceived challenges and opportunities, perceptions of their role in animal welfare, public health, and One Health contexts. Interviews were documented through detailed written notes taken during and immediately after each session. The collected information was subsequently organized into a structured data matrix using Microsoft Excel version 365 (Microsoft Corporation, Redmond, WA, USA) to enable systematic comparison across organizations (see Supplementary Material S2). Interviews lasted between 20 and 45 min, with an average duration of approximately 30 min. All interviews were conducted in Spanish by the same researcher (A.S.R.P.), which contributed to consistency in the application of the interview guide across participants.

### 2.4. Data Analysis

Data analysis was conducted in two complementary stages.

#### 2.4.1. Descriptive Analysis

A descriptive analysis was performed to characterize the identified organizations by geographic distribution, institutional affiliation (public vs. private), organizational structure, leadership, activities, and reported outputs (e.g., number of animals assisted, sterilizations, adoptions, and educational activities). Continuous variables were summarized as medians/IQR, while categorical variables were summarized as frequencies and proportions. This analysis also enabled estimation of the proportion of universities that host animal welfare organizations at the national level.

#### 2.4.2. Framework-Informed Analysis

Interview data were analyzed using a framework-informed approach [17,18], in which analytical dimensions were defined a priori based on key components of free-roaming dog and cat population management and One Health principles, rather than derived inductively from the data. Interview notes, taken during and immediately after each session, were reviewed and expanded by the same researcher immediately afterward to minimize information loss and ensure completeness. The notes were then independently reviewed by a second researcher (S.D.B.A.) to verify that the recorded information accurately reflected the content of each session. Subsequently, the notes were systematically organized within each analytical dimension using a structured data matrix (see Supplementary Material S2), enabling systematic comparison across organizations. Discrepancies or ambiguities in interpreting responses were discussed and resolved by consensus within the research team. This process was considered adequate to ensure consistency in data interpretation, given the applied and exploratory scope of the study and the use of a structured interview guide that constrained the range of responses.

It is acknowledged that the use of detailed written notes rather than verbatim transcripts limits the depth of qualitative analysis, including potential gaps in nuance and the impossibility of applying procedures such as member-checking to a complete textual record. These limitations are discussed further in Section 5.

This approach was considered appropriate given the applied and exploratory nature of the study and the use of a structured interview guide that ensured consistency across participants. The four dimensions were:

- Organizational Capacity (e.g., membership, leadership structure, continuity of activities);
- Animal Care and Population Management Activities (e.g., rescue, sterilization, adoption, feeding, and veterinary interventions);
- Public Health and Community Engagement (e.g., vaccination, parasite control, educational activities, awareness campaigns);
- Institutional Integration and Sustainability (e.g., formal recognition, academic support, links to veterinary programs, funding mechanisms).

### 2.5. Ethical Considerations

This study did not require approval from an institutional ethics committee. Data collection was conducted through two sources: publicly available information retrieved from official university websites and social media platforms, and semi-structured interviews focused exclusively on organizational-level information. No personal data was collected from individual participants, and no sensitive information of any kind was obtained or processed. Interview participants were informed of the study objectives and the intended academic use of the information prior to each session and provided their consent for its use. Given the nature of the data collected, the study was deemed exempt from formal ethical review in accordance with institutional guidelines.

## 3. Results

### 3.1. Identification and Distribution of University Animal Welfare Organizations

**Figure 1:**
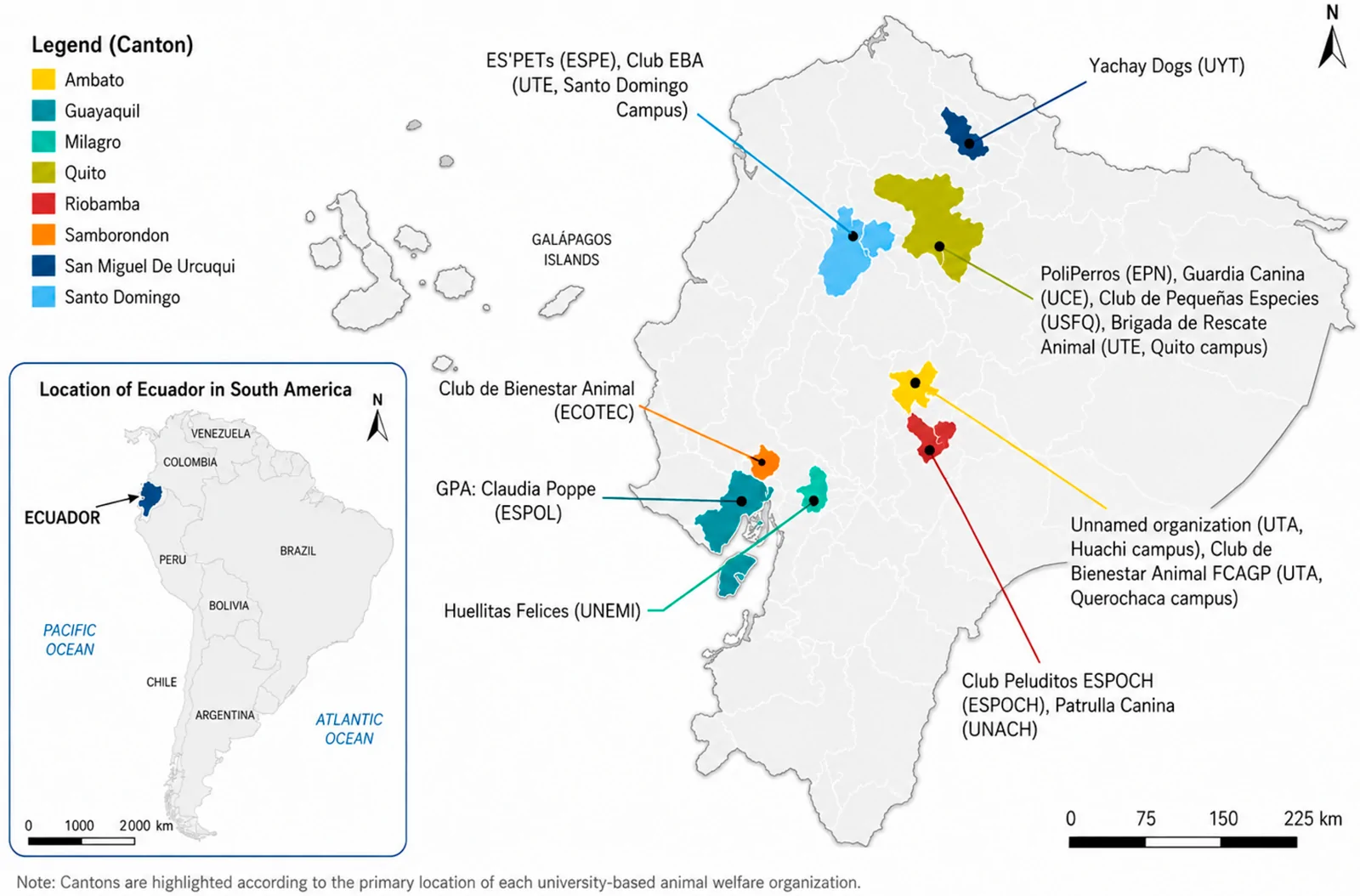
Geographic distribution of the identified organizations in Ecuadorian Universities.

A total of 63 universities and polytechnic schools officially recognized by CACES were analyzed. Among these institutions, 12 universities (19.0%) hosted animal welfare organizations, corresponding to 14 groups, with two universities each hosting two organizations. Geographically, five organizations are located in the Coastal region and nine in the Sierra region, with none in the Amazon or Insular regions. The identified organizations include Yachay Dogs (Universidad Yachay Tech, Urcuquí), PoliPerros (Escuela Politécnica Nacional, Quito), Club Peluditos ESPOCH (Escuela Superior Politécnica del Chimborazo, Riobamba), GPA: Claudia Poppe (Escuela Superior Politécnica del Litoral, Guayaquil), Guardia Canina (Universidad Central del Ecuador, Quito), *ES’PEts* (Universidad de las Fuerzas Armadas ESPE, Santo Domingo), Club de Pequeñas Especies (Universidad San Francisco de Quito, Quito), Huellitas Felices (Universidad Estatal de Milagro, Milagro), an unnamed organization and Club de Bienestar Animal FCAGP (Universidad Técnica de Ambato, Ambato), Brigada de Rescate Animal and Club EBA (Universidad UTE, Quito and Santo Domingo), Patrulla Canina (Universidad Nacional de Chimborazo, Riobamba), and Club de Bienestar Animal (Universidad ECOTEC, Samborondón). Among the universities hosting these organizations, nine are public, and three are private. Most organizations (12/14) primarily work with dogs, while two focus on cats. Additionally, 11 out of 12 organizations reported caring for animal populations located on or near their campuses, with an average population size of 26.3 animals (range: 5–90). Five organizations (35.7%) are affiliated with veterinary or animal science programs. The organizations were founded between 2009 and 2025, although information on two groups was unavailable. Detailed information is presented in Table 1, while Figure 1 illustrates their geographic distribution. A complete list of screened universities is provided in Supplementary Material S1.

### 3.2. Organizational Structure and Institutional Integration

Interviews were conducted with representatives from 12 of the 14 identified organizations, enabling a more detailed characterization of their structure and functioning. On average, organizations have been active for 7.3 years (range: 1–17 years) and have 16 active members (range: 2–40). While all organizations originated as student-led initiatives, their current leadership structures vary: six are led by students, three by faculty members, and three by university administrative staff. Most organizations (9/12) are formally recognized as student clubs or groups within their universities. However, only three reported having formal bylaws or statutes defining their mission, vision, and operational guidelines. Faculty involvement is common, with 10 out of 12 organizations receiving some level of academic guidance or mentorship.

### 3.3. Animal Care and Management Activities

The primary activities reported by these organizations revolve around direct animal care and population management. Over the past 12 months, organizations reported a median of 20 animals treated through interventions such as vaccination, deworming, sterilization, rescues, feeding, and general veterinary care (IQR: 5–40; n = 11 organizations with available data). One organization—the *Club de Pequeñas Especies* at Universidad San Francisco de Quito—reported treating approximately 2000 animals through 11 vaccination and deworming campaigns, representing an extreme outlier; given this distributional asymmetry, median and IQR are reported throughout this section. Regarding sterilization, organizations reported a median of 5 procedures in the past year (range: 3–50; n = 7). Adoption efforts are also a key component of their work, with a median of 3 successful adoptions over the past 12 months (IQR: 0–5; n = 9).

### 3.4. Education, Outreach, and Training

Beyond direct animal care, many organizations engage in educational and outreach activities. Seven out of 12 organizations reported conducting activities such as workshops, training sessions, or awareness campaigns related to animal welfare and responsible ownership. In organizations affiliated with veterinary or animal science programs (at USFQ, UTA, UTE Quito, UTE Santo Domingo, and ECOTEC), participation also contributes to student training through pre-professional internships, highlighting their role in experiential learning.

### 3.5. Funding Mechanisms and Resource Constraints

None of the organizations reported receiving direct financial support from their host universities. All organizations rely on donations from members of the university community. Additionally, 10 out of 12 generate income through fundraising activities such as the sale of food or merchandise. Only two organizations reported receiving support from corporate sponsors, and one reported collecting monthly membership fees.

### 3.6. Thematic and Framework Analysis

Across organizations, founding narratives converged on two interrelated drivers: direct exposure to animal abandonment on university campuses and a personal commitment to animal welfare among founding members. Most organizations emerged not through institutional planning but through the spontaneous response of small groups of students—often just two or three—who encountered abandoned or free-roaming animals and acted without formal support. Representatives frequently described these beginnings as highly challenging, marked by large numbers of distressed animals and limited community awareness. In some cases, the situations were especially difficult, including reports of animals being deliberately abandoned on campus grounds tied in sacks (e.g., Universidad Central del Ecuador). Alongside these situational triggers, a shared concern for animal welfare was consistently cited as a motivating factor, with some groups explicitly referencing frameworks such as the Five Freedoms (e.g., Universidad UTE—Campus Santo Domingo). Over time, many organizations evolved from informal student initiatives into more structured entities, sometimes prompted by the graduation of founding members and the resulting need for continuity. In one case, this process also took on a commemorative dimension: the organization was named after one of its founders, who was killed alongside her mother, thereby turning the group into both a welfare initiative and a tribute to her legacy (e.g., Escuela Politécnica del Litoral).

#### 3.6.1. Organizational Capacity

The organizational capacity of university-based animal welfare organizations showed considerable internal diversity in both governance structures and continuity over time. Leadership models ranged from formally differentiated roles—such as feeding, hydration, walking, and communications—to explicitly horizontal structures that rejected hierarchical titles. As one representative explained, “The club operates through commissions under horizontal leadership. We do not like distinctions such as president, vice-president, or similar titles” (GPA: Claudia Poppe). In some organizations, particularly those with smaller student bodies, coordination gradually shifted from students to faculty or administrative staff.

A recurring challenge across organizations was sustaining volunteer engagement. Participation often declined over the academic cycle, and the graduation of founding or core members frequently led to losses in institutional memory and operational capacity. One representative noted: “There are often problems organizing volunteers. By the end of projects or activities, very few remain, and sometimes we fail to meet our objectives” (Yachay Dogs). This suggests that volunteer turnover is one of the main structural vulnerabilities of these groups, affecting both the scale and continuity of their interventions. Some organizations have addressed this issue through formal incentive mechanisms, such as volunteer recognition systems and membership fees intended to strengthen commitment and a sense of belonging (e.g., Club de Pequeñas Especies, USFQ).

Regarding resource management, self-funding through community-based fundraising was universal, and no organization reported direct financial support from its host institution. As one representative stated, “All the financial resources we need for our activities are obtained through self-management” (Club Peluditos ESPOCH). Despite these constraints, several organizations had developed systematic practices such as animal registration with municipal authorities and structured record-keeping, suggesting that limited funding does not necessarily preclude organizational formalization.

#### 3.6.2. Animal Care and Population Management Activities

Animal care and population management activities ranged from basic daily care to technically structured protocols. At the more developed end, some organizations implemented integrated systems including rescue protocols, external veterinary care, sterilization, vaccination, deworming, behavioral management, and GPS-based monitoring of campus animal populations, sometimes with support from ethologists or trainers. These groups also operated within formal regulatory frameworks and assumed legal responsibility for incidents involving campus animals under municipal ordinances. In contrast, other organizations focused mainly on feeding, basic health monitoring, and facilitating adoptions, reflecting differences in available resources and institutional support.

A consistent challenge across organizations was the low rate of successful adoptions. This appeared to be a structural feature of campus-based animal management, shaped in part by the transient nature of university communities: “It is complicated for people to adopt dogs. It tends to be graduating students who take the dog they have grown attached to” (Yachay Dogs). This suggests that adoption alone is insufficient as a population management strategy in these settings.

In response, some organizations adopted alternative management approaches. Most notably, Trap–Neuter–Return (TNR) was reported as a primary strategy for managing feral cat colonies in at least one institution: “Apart from trying to place animals for adoption, over the years, a philosophy we have strongly promoted is Capture, Sterilize, and Release. It has been an ethical and quite effective way of managing the cat colonies at ESPOL” (GPA: Claudia Poppe). This illustrates the incorporation of internationally recognized management strategies even in resource-constrained settings.

The scope of activities also extended beyond direct animal care. Some organizations developed educational and training programs, including workshops and inter-institutional campaigns, whereas others positioned themselves mainly as support nodes for external rescue foundations rather than frontline rescue groups. This suggests an emerging, though informal, functional differentiation within the university animal welfare ecosystem.

#### 3.6.3. Public Health and Community Engagement

Public health and community engagement activities showed that campus animal populations were closely linked to surrounding urban and peri-urban communities. Several organizations reported that free-roaming animals migrated onto campuses from nearby areas due to neglect, abandonment, or proximity to waste-disposal sites. As one representative explained, “In the surrounding communities, there are many families with pets who unfortunately do not care for them well… those dogs have migrated to our campus in search of food, affection, and shelter… If we manage to place 5 in adoption, another 5 come in to take their place” (Yachay Dogs).

Sterilization, often implemented through Trap–Neuter–Return approaches, was the intervention most frequently associated with reductions in reproductive activity and campus animal numbers. Some organizations also reported using chemical immobilization to sterilize feral or highly defensive animals. Community engagement extended beyond campus boundaries in several cases. Some organizations reported outreach activities in rural communities, often coordinated with parish-level GADs to support free veterinary campaigns for dogs and cats. These activities were also described as opportunities for practical training of veterinary students (Club de Pequeñas Especies, USFQ). Lastly, some organizations reported formal adoption protocols that included post-adoption follow-up through periodic contact with adopters and monitoring of animal welfare outcomes. One representative described requiring that adopters own their homes, reside in the city, and provide regular updates after adoption (UTE Brigada de Rescate Animal).

#### 3.6.4. Institutional Integration and Sustainability

Institutional support across organizations was predominantly material rather than financial. Physical spaces—storage areas, cooking facilities, and dedicated kennels—constituted the most common form of provision, complemented in some cases by indirect logistical support from university security personnel. None of the organizations reported receiving direct financial contributions from their host institutions.

Formal recognition varied considerably. In one case, the continued presence of campus animals was debated at the highest institutional governance level, requiring the organization to prepare a formal strategic plan to prevent the removal of resident animals: “The issue of the dogs has reached the highest level of governance, having been debated in sessions of the Politécnico Council to ensure their permanence in the face of attempted eviction by faculty members who oppose their presence on campus” (PoliPerros). At the other end of the spectrum, at least one organization operated without any formal recognition, relying entirely on self-managed resources: “All activities are self-managed; many times we even have to contribute money from our own pockets when donations are insufficient” (unnamed organization, UTA Huachi Campus).

Several organizations reported functional partnerships beyond the university. Municipal sterilization programs, vaccine provision through university veterinary departments, and food collection coordinated through student welfare offices were recurring forms of external support. One organization described an informal division of roles with municipal waste management workers: “We, primarily as veterinary professionals and students, try to look after their health, while the EMASEO workers take care of feeding them” (Brigada de Rescate Animal, UTE Quito Campus).

One organization reported an environmental dimension to its campus animal management activities. Representatives noted that maintaining a sterilized and cared-for cat population reduced the likelihood of feral behavior and predation on wildlife in an adjacent protected forest: “Keeping the cat population controlled—through feeding, veterinary care, and sterilization—helps prevent them from becoming feral and predating the wildlife of this protected forest” (GPA: Claudia Poppe).

## 4. Discussion

This study provides the first national-level characterization of university-based animal welfare organizations in Ecuador and highlights their emerging yet largely underrecognized role in managing free-roaming dog and cat populations within a One Health framework. The findings suggest that these organizations represent a distributed, community-embedded network with significant operational potential, yet constrained by structural and institutional limitations.

### 4.1. University-Based Organizations as Decentralized Actors in Animal Population Management

The identification of animal welfare organizations across multiple universities indicates that these groups are not isolated initiatives but rather a widespread phenomenon within the Ecuadorian higher education system. Their activities—particularly rescue, sterilization campaigns, adoption programs, and educational outreach—align closely with internationally recognized strategies for humane dog population management. The activities reported by the organizations in this study directly contribute to several of these impact domains, suggesting that—even in the absence of formal integration into public policy—they are already influencing key indicators of population management.

However, ICAM emphasizes that responsibility for dog population management should primarily rest with governmental authorities—a principle that also applies to cat population management—with non-governmental actors playing a complementary role [4,5]. The findings of this study reveal a partial inversion of this principle in the Ecuadorian context, where university-based and civil society organizations often assume operational responsibilities in the absence of sustained governmental leadership.

The absence of identified organizations in the Amazonian and Insular regions warrants consideration. Geographically, both regions host a very limited number of CACES-recognized universities relative to the Sierra and Costa, which concentrate the structural preconditions for student-led initiatives—namely, larger student populations and greater institutional density. In the Insular region specifically, the management of free-roaming dogs and cats is closely regulated under the Galápagos National Park conservation policy, which may reduce the operational space available to informal student organizations and shift responsibility toward institutional actors. Additionally, the systematic search conducted in this study relied on publicly available digital information; regions with lower internet connectivity or less active institutional social media presence may be underrepresented in the findings. Whether the absence of organizations in these regions reflects a true gap in university-based animal welfare activity or a limitation of the search strategy cannot be determined from the available data, and future studies should consider direct outreach to universities in these regions to complement online mapping approaches.

### 4.2. Contributions to One Health: Bridging Animal Welfare and Public Health

Animal welfare has been identified as a fundamental component of One Health, as improving animal health and living conditions can reduce the circulation and transmission of pathogens [19]. In this context, the activities of university-based organizations—particularly sterilization, vaccination, and community education—may represent indirect contributions to disease prevention, though their public health impact has not been directly assessed in this study.

This connection is further supported by evidence from Ecuador, where sterilization campaigns organized by animal welfare groups have served as platforms for zoonotic disease surveillance. A recent study we conducted demonstrated that collaboration between academic researchers and animal organizations facilitated the collection of hundreds of biological samples from free-roaming dogs, significantly reducing logistical and economic barriers to research [20,21,22]. This model suggests that animal welfare interventions could potentially be leveraged to generate epidemiological data, positioning these organizations as candidate partners in One Health research.

Importantly, such collaborations are particularly valuable in low- and middle-income countries, where research funding and infrastructure are limited. In Ecuador, a very limited budget allocated to scientific programs makes community-based approaches essential for advancing surveillance efforts and epidemiological research. The organizations identified in this study, therefore, represent not only service providers but also could potentially contribute as nodes within decentralized surveillance systems to promote One Health-oriented research.

### 4.3. Operational Capacity and Structural Limitations

Despite their contributions, the organizations analyzed in this study face significant challenges that limit their impact and sustainability. Most operate on a volunteer basis, with very limited financial resources, high member turnover, and variable levels of institutional support. From a systems perspective, these limitations directly affect their operational capacity, a key dimension of effective population management. The absence of stable funding and formal recognition restricts their ability to scale up activities such as sterilization campaigns, which are essential for achieving long-term population control.

As noted above, ICAM guidelines call for coordinated, sustained interventions supported by governance structures and financing mechanisms [4,5]. The fragmented and largely informal nature of university-based organizations contrasts with these recommendations, highlighting a gap between current practice and optimal system design. At the same time, their embeddedness within universities offers unique opportunities. Access to students, academic resources, and potential interdisciplinary collaboration positions these organizations as strategic platforms for integrating education, research, and community engagement, especially when veterinary schools are involved. However, this potential remains underutilized in many cases due to the lack of formal institutional integration.

### 4.4. Contextual Factors Influencing the Predominance of Dog-Related Activities in Ecuadorian University Organizations

The predominance of dog-related activities among the university-based animal welfare organizations identified in this study may reflect broader patterns of companion animal ownership and abandonment in Ecuador, although this interpretation should be made with caution. At present, there is no nationwide exploratory study quantifying the number of free-roaming dogs and cats in Ecuador, which limits direct comparisons between species. However, data from the 2022 Ecuadorian National Census provide an indirect indication of household preferences. According to a report by the National Institute of Statistics and Census (INEC), 1,326,537 households with children under 12 years of age reported owning dogs or cats; among these households, 730,435 reported owning only dogs, whereas 146,484 reported owning only cats [23]. Although these figures refer to owned animals within households rather than free-roaming populations, they suggest that dogs may be more prevalent in Ecuadorian households than cats. This tendency could contribute, at least in part, to the greater visibility of dog abandonment and to the prioritization of dog-related activities by university-based animal welfare organizations.

At the same time, the observed emphasis on dogs may also reflect contextual and organizational factors. Free-roaming dogs are often more visible in urban and peri-urban public spaces, where they may raise community concerns about abandonment, traffic accidents, bites, waste management, and zoonotic disease risks [24,25,26,27,28]. These factors may encourage university-based organizations to direct their efforts toward dog rescue, sterilization campaigns, veterinary care, and community education. Nevertheless, because this study did not assess the actual proportion of free-roaming dogs and cats, it cannot determine whether the predominance of dog-focused activities is driven primarily by national population dynamics, by the organizations’ strategic choices, or by a combination of both. Future research incorporating systematic population estimates of free-roaming dogs and cats would be necessary to clarify these relationships and to inform more targeted population management strategies.

### 4.5. Toward Integration Within Institutional and Policy Frameworks

The findings of this study suggest that strengthening the impact of university-based animal welfare organizations will require greater alignment with institutional and governmental structures. Rather than replacing state responsibility, these organizations could be integrated into coordinated national or municipal strategies for animal population management. The One Health approach explicitly calls for interdisciplinary collaboration across sectors, including academia, public health, veterinary services, and community organizations [8]. University-based groups are uniquely positioned to facilitate such collaboration, acting as intermediaries between local communities and formal institutions. Furthermore, education and awareness are central to improving animal welfare and public health outcomes. The strong involvement of students in these organizations presents an opportunity to foster a new generation of professionals trained in One Health principles and with practical experience in community-based interventions.

## 5. Conclusions and Implications

University-based animal welfare organizations in Ecuador operate as decentralized, resource-constrained initiatives that nonetheless generate measurable outcomes in sterilization, adoption, and veterinary care. Their activities align structurally with community-based One Health approaches, particularly in integrating animal population management with zoonotic disease surveillance and environmental health. The findings suggest that formal institutional recognition, inter-sectoral partnerships, and dedicated funding mechanisms are the primary conditions required to enhance the sustainability and scalability of these efforts. Integrating these organizations into broader governance frameworks is a viable, low-cost strategy to advance animal welfare and public health goals in Ecuador and comparable low- and middle-income settings.

## 6. Limitations of the Study

This study has limitations that should be considered when interpreting the findings. First, the identification of university-based animal welfare organizations relied primarily on publicly available information from social media platforms and official university websites. As a result, only organizations with evidence of activity updated through 2026 were included, while groups lacking recent online presence were excluded. This constitutes a potential source of selection bias, as organizations with active digital visibility may differ systematically from those without it in terms of size, institutional recognition, or resource availability. This approach may have led to an underestimation of the true number of organizations, particularly those that are inactive online but still operational at a local level. Second, the variability in the type, depth, and accuracy of publicly reported information limited the ability to systematically assess organizational structure, scale of activities, and impact. Third, the study’s cross-sectional design does not capture temporal dynamics, such as the creation, dissolution, or fluctuations in these organizations’ activity levels over time. Additionally, given the absence of direct engagement with all identified groups, some findings may not fully reflect internal processes, challenges, or achievements, highlighting the need for future studies that incorporate primary data collection methods, such as surveys or other interviews.

The use of detailed written notes rather than verbatim transcripts represents a methodological limitation that warrants explicit consideration. Verbatim transcripts would have allowed for more fine-grained analysis of language and meaning, facilitated member-checking procedures, and provided a more complete audit trail for qualitative interpretation. In this study, the absence of full transcripts means that certain nuances in participants’ responses may not have been fully captured, and that the depth of the framework-informed analysis is necessarily constrained by the granularity of the recorded data. To mitigate these limitations, notes were taken by the same researcher throughout all interviews using the structured interview guide as a reference framework and were reviewed and expanded immediately after each session to minimize information loss. A co-author independently reviewed the notes prior to analysis, and interpretive discrepancies were resolved by consensus. Given the applied and exploratory scope of the study, and the fact that the analytical framework was defined a priori rather than derived inductively from the data, these measures were considered adequate to support the cross-organizational comparisons reported. Future studies should consider audio recording and full transcription to enable deeper qualitative analysis and enhance methodological rigor.

Finally, the sampling strategy employed for the interviews warrants critical consideration. Participants were selected from among the organizations identified through the institutional mapping process, based on their willingness to participate and the availability of a representative with sufficient knowledge of their organizational activities. This constitutes a purposive convenience sample, which, while appropriate for exploratory qualitative research, limits the extent to which the interview-based findings can be generalized beyond the participating organizations. In particular, organizations that declined to participate or did not respond may differ systematically from those that did—for instance, in terms of institutional recognition, organizational maturity, or openness to external scrutiny. The interview-based findings should therefore be interpreted as illustrative of the range of experiences and organizational models present in the Ecuadorian university context, rather than as representative of all university-based animal welfare organizations in the country.

## Figures and Tables

**Table 1:** Animal Welfare Organizations identified in universities in Ecuador. The complete information collected through web searches and interviews is available in Supplementary Materials S1 and S2.

n°	Organization Name	Year Founded	University	Acronym	Public/Private	Canton	Province	Veterinary Affiliation (Yes/No)
1	Yachay Dogs	2016	Universidad Yachay Tech	UYT	Public	Urcuquí	Imbabura	No
2	Poliperros	2013	Escuela Politécnica Nacional	EPN	Public	Quito	Pichincha	No
3	Club Peluditos Espoch	2022	Escuela Superior Politécnica de Chimborazo	ESPOCH	Public	Riobamba	Chimborazo	No
4	GPA: Claudia Poppe	2009	Escuela Superior Politécnica del Litoral	ESPOL	Public	Guayaquil	Guayas	No
5	Guardia Canina	2016	Universidad Central del Ecuador	UCE	Public	Quito	Pichincha	No
6	ES’PEts	?	Universidad de las Fuerzas Armadas ESPE	ESPE	Public	Santo Domingo	Santo Domingo de los Tsáchilas	No
7	Club de Pequeñas Especies	2023	Universidad San Francisco de Quito	USFQ	Private	Quito	Pichincha	Yes
8	Huellitas Felices	?	Universidad Estatal de Milagro	UNEMI	Public	Milagro	Guayas	No
9	unnamed organization	2022	Universidad Técnica de Ambato	UTA (Huachi campus)	Public	Ambato	Tungurahua	No
10	Club de Bienestar Animal FCAGP	2018	Universidad Técnica de Ambato	UTA (Querochaca campus)	Public	Ambato	Tungurahua	Yes
11	Brigada de Rescate Animal	2020	Universidad UTE	UTE (Quito campus)	Private	Quito	Pichincha	Yes
12	Club EBA	2024	Universidad UTE	UTE (Sto. Domingo campus)	Private	Santo Domingo	Santo Domingo de los Tsáchilas	Yes
13	Patrulla Canina	2016	Universidad Nacional de Chimborazo	UNACH	Public	Riobamba	Chimborazo	No
14	Club de Bienestar Animal	2025	Universidad Tecnológica ECOTEC	ECOTEC	Private	Samborondón	Guayas	Yes

## Data Availability

The data supporting the reported results are partially available. The institutional mapping dataset and organizational characterization data are provided as Supplementary Materials (S1 and S2) accompanying this article. Raw interview data are not publicly available due to privacy considerations and the confidentiality commitments made to participants during data collection. Anonymized summaries of interview responses are reflected in the findings reported in the manuscript. Further inquiries can be directed to the corresponding author.

## Supplementary Materials

The following supporting information can be downloaded at: https://www.mdpi.com/article/10.3390/ani16182885/s1, Supplementary Material S1: List of the 63 officially recognized universities and polytechnic schools included in the institutional mapping, Supplementary Material S2: Details of the 14 university animal welfare organizations identified, and Supplementary Material S3: Informed Consent Form of interviews.
